# Non-transport functions of motor proteins in corticogenesis

**DOI:** 10.3389/fcell.2026.1843278

**Published:** 2026-08-11

**Authors:** Natalia Mitina, Alexandra Medyanik, Victor Tarabykin, Elena Kondakova

**Affiliations:** 1 Laboratory of Genetics of Brain Development, Institute of Neurosciences, Lobachevsky State University of Nizhny Novgorod, Nizhny Novgorod, Russia; 2 Department of Genetics and Life Sciences, Sirius University of Science and Technology, Sochi, Russia

**Keywords:** cortical development, dynein, kinesin, multipolar-to-bipolar transition, myosin, neural progenitor proliferation, neurite outgrowth, neuronal migration

## Abstract

Cerebral cortex development is a tightly coordinated sequence of interconnected processes: proliferation of neural progenitors, neuronal migration, neurite differentiation, axonal pathfinding, and synaptogenesis. Traditionally, motor proteins–dyneins, kinesins, and myosins–have been viewed as molecules mediating intracellular transport along the cytoskeleton. Nevertheless, data accumulated over the past decade provide compelling evidence for a fundamentally different, non-transport role of these proteins in nervous system development. This review systematizes current understanding of the non-transport functions of motor proteins at key stages of corticogenesis. We review the molecular mechanisms that enable dyneins, kinesins, and myosins to function as regulators of cortical development. We focus specifically on the causal relationship between disruptions to the non-transport functions of motor proteins and cortical developmental disorders, including microcephaly, lissencephaly, and agenesis of the corpus callosum.

## 1 Introduction

The development of the cerebral cortex is a multistage, precisely coordinated process that ensures the formation of the six-layered structure of the neocortex. Stage and cell-type-specific genetic programs regulate the balance between proliferation of neural stem cells, cell cycle exit, and subsequent cell fate specification and neuronal differentiation. Precise regulation of cell cycle duration and the proportion of cells exiting it define the ultimate cortical size and the proportions of distinct cell types (Sun and Hevner, 2014; Subramanian et al., 2017). As differentiation proceeds, postmitotic neurons migrate toward the cortical plate and establish cortical layers in an “inside-out” fashion (Ohtaka-Maruyama and Okado, 2015). In the developing neocortex, this process coincides with dendrito- and axonogenesis, elaborate dendrites and an axon. Subsequently, upon reaching their final position within the cortex, neurons form synaptic connections and integrate into neural circuits (Gavrish et al., 2024). Thus, corticogenesis constitutes a sequential progression of proliferation, migration, neurite differentiation, and synaptogenesis. Disruption in any of these stages result cortical malformations commonly known as neurodevelopmental disorders (NDDs) (Guerrini and Dobyns, 2014; Sun and Hevner, 2014).

The formation of cortical cytoarchitecture during corticogenesis is mediated by dynamic cytoskeletal remodeling and the coordinated activity of molecular motors. The cytoskeleton, a dynamic network of microtubules, actin filaments, and intermediate filaments, plays a pivotal role in these processes. On one hand, it defines cell architecture, and on the other hand, it also serves as a scaffold for intracellular transport (Achkasova et al., 2026).

Traditionally, motor proteins such as dyneins, kinesins, and myosins have been regarded mostly as key effectors of intracellular transport, conveying vesicles, organelles, mRNAs, and protein complexes along cytoskeletal “tracks” (Hirokawa et al., 2010). In neurons, with their polarized structure and extremely long axons and dendrites, the transport function of motor proteins is of critical importance: kinesins predominantly mediate anterograde transport toward the plus-ends of microtubules, whereas cytoplasmic dynein is responsible for retrograde cargo movement toward the cell body (Roberts et al., 2013; Konjikusic et al., 2021; Rao and Gennerich, 2024). Myosins, in turn, carry out local short-range transport in actin-rich domains such as axonal growth cones, presynaptic terminals, and postsynaptic spines (Bridgman, 2004; Pollard, 2020). Mutations in motor protein genes lead to severe neurodevelopmental pathologies, including various malformations of cortical development (Maillard et al., 2023).

However, over the past decades, a substantial body of evidence has been accumulated that indicates that these molecules have other functions beyond intracellular transport (Muresan and Muresan, 2012). Studies linking mutations in the *LIS1*, *Nde1*, and *Ndel1* genes with lissencephaly and severe forms of microcephaly have provided evidence of the involvement of cytoplasmic dynein 1 in processes other than vesicular transport (Alkuraya et al., 2011; Monda and Cheeseman, 2018; Tsai et al., 2024). Similarly, kinesin KIF1A, originally described as a transporter of synaptic vesicle precursors, has proven to be critically important for interkinetic nuclear migration in radial glial cells, and its mutations are associated not only with neuropathies but also with defects in neurogenesis (Okada et al., 1995; Falnikar et al., 2024). Myosin II, particularly isoform IIB, has been shown to play an important role in cell migration, adhesion, cytokinesis, and plasticity of postsynaptic dendritic spines. Point mutations in the part of the gene encoding the motor domain cause disruption of these processes (Newell-Litwa et al., 2015; Dantas et al., 2016).

Of particular note is the fact that certain kinesins, such as members of the M-kinesin subfamily (KIF2A, KIF2B, KIF2C), are not involved in cargo transport at all but instead specialize in microtubule depolymerization, fulfilling essential roles in the regulation of microtubule length and the organization of the mitotic spindle (Manning et al., 2007; Konjikusic et al., 2021).

The aim of the present review is to systematize current knowledge regarding the non-transport functions of motor proteins in corticogenesis. We examine their involvement in the key stages of corticogenesis: proliferation and polarization of neural progenitors; neuronal migration; axonal and dendritic outgrowth; and the formation of synaptic contacts. Particular emphasis is on the underlying molecular mechanisms and the relationship between disruptions in non-transport functions of motor proteins and pathologies of cortical development.

## 2 Motor proteins. Types and functions of motor proteins

**FIGURE 1:**
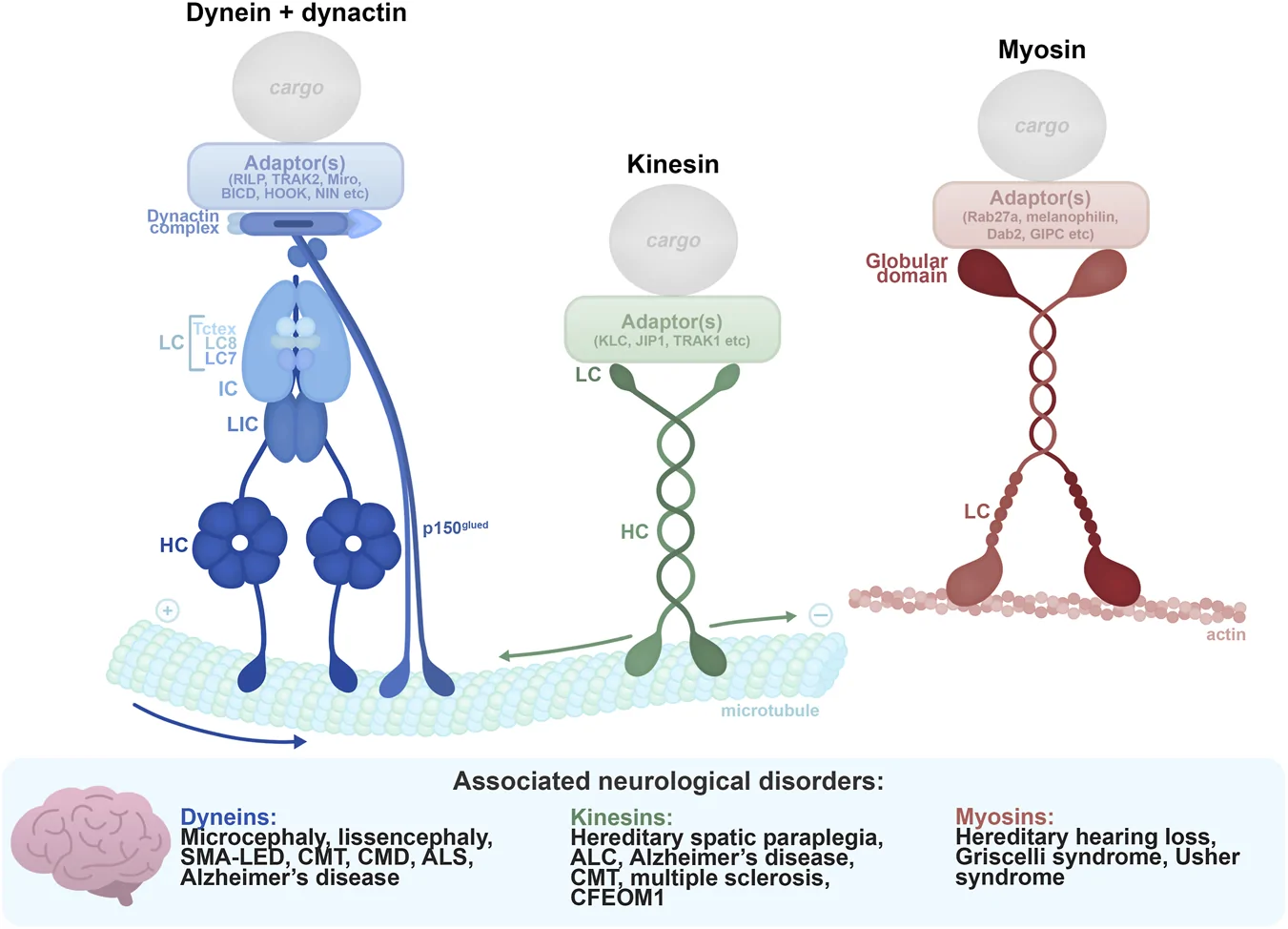
General structural organization of motor proteins. Schematic representations of the dynein-dynactin complex, kinesin and myosin highlight their overall domain architecture and characteristic structural features. Adaptors link motor to cargo, determine cargo specificity and regulate motor activity. Selected neurodevelopmental and neurodegenerative disorders associated with their dysfunction are listed under each motor protein.

Motor proteins are molecular motors capable of moving along the cytoskeleton, comprising three superfamilies: dyneins, kinesins, and myosins (Figure 1) (Yang et al., 2015).

Molecular motors are crucial for neuronal function, development, and survival. They mediate intracellular transport of essential components, including synaptic vesicle precursors, neurotransmitters, and neurotropic factor receptors, and RNA within axons, dendrites, and synapses. Moreover, non-transport roles of molecular motors have been identified in regulating central and peripheral nervous system development (Hirokawa et al., 2010). Beyond transport itself, motor proteins participate in the organization of cellular mechanics and in mechanotransduction processes. Mechanical forces are generated primarily through the polymerization of actin filaments and actomyosin contractility driven by myosin motors, with additional contributions from microtubule polymerization and dynein-mediated sliding (Pillai and Franze, 2024). Motor protein dysfunction is directly linked to the pathogenesis of brain developmental disorders and neurodegenerative diseases (Hirokawa et al., 2010).

### 2.1 Dyneins

Dyneins are mechanoenzymes that move along microtubules using ATP hydrolysis, with their activity directed toward the minus-end of microtubules. Cytoplasmic dynein comprises a large protein complex consisting of several polypeptide subunits:

- Two heavy chains (∼520 kDa) with ATPase activity that mediate movement along microtubules;
- Two intermediate chains (∼74 kDa);
- Four light intermediate chains (∼33–59 kDa);
- Several light chains (∼10–14 kDa).

Proteins of the dynein superfamily are divided into three major groups: cytoplasmic dyneins, axonemal dyneins, and intraflagellar dyneins (IFT). The axonemal dyneins are also known as ciliary or flagellar dyneins (Hirokawa et al., 2010; Romero et al., 2023; Mukhopadhyay et al., 2024).

Furthermore, cytoplasmic dynein possesses an important associated protein complex–dynactin. It comprises p150Glued, p62, dynamitin (p50), Arp1 (actin-related protein 1), CAPZα and CAPZβ, p27, and p24. Dynactin plays a crucial role in regulating dynein activity and enables its stable binding to transported cargo (Hirokawa et al., 2010; Yang et al., 2015).

For proper function, the dynein-dynactin complex requires additional cofactors called activators. Several dynein motility activators have been identified, including LIS, proteins of the BICD family (BICDR1 and BICDR2 in mammals), proteins of the HOOK family (HOOK1, HOOK2, and HOOK3), RAB11-FIP3, Spindly, Ninein (NIN), and NIN-like proteins (NINL) (Splinter et al., 2012; Elshenawy et al., 2019; Olenick et al., 2019; Tempes et al., 2020; Reimer et al., 2023).

Another group of proteins, called adapters, mediates dynein binding to cargo. Adapters include Rab7-interacting lysosomal protein (RILP), trafficking kinesin protein 2 (TRAK2), and mitochondrial Rho-GTPase 1 (Miro). The dynein-dynactin complex also utilizes other regulatory partners, including microtubule plus-end tracking proteins (+TIPs) such as end-binding protein 3 (EB3), cytoplasmic linker protein 170 (CLIP-170), the LIS1 protein, and scaffolding proteins (NUDEL and NUDE) (Jordens et al., 2001; MacAskill et al., 2009; Van Spronsen et al., 2013; Wang et al., 2013; Baumbach et al., 2017; Tempes et al., 2020).

Cytoplasmic dynein plays a key role in cellular dynamics. It mediates transport of membrane vesicles toward microtubule minus-ends and participates in cell division processes: contributing to spindle formation, centrosome separation, nuclear envelope breakdown, and chromosome segregation during mitosis. Defects in cytoplasmic dynein and its associated dynactin complex lead to the development of several severe neurological diseases, including amyotrophic lateral sclerosis (ALS), lissencephaly, Alzheimer’s disease, and other disorders (Sharp et al., 2000; Chen et al., 2014; Yang et al., 2015; Markus et al., 2020).

Dynein dysfunction also underlies many cases of cortical malformations, congenital muscular dystrophy (CMD), and Charcot-Marie-Tooth disease (CMT). Heterozygous mutations in the dynein heavy chain gene (*DYNC1H1*) have been identified in patients with cortical malformations ranging from microcephaly with dysgyria to lissencephaly, as well as in peripheral neuropathies such as spinal muscular atrophy with lower extremity predominance (SMA-LED) (Marzo et al., 2019; Romero et al., 2023).

### 2.2 Kinesins

Proteins of the kinesin superfamily (KIF) are mechanoenzymes responsible for motility directed toward both the plus- and minus-ends of microtubules, as well as for their depolymerization. KIFs are classified into three major groups based on the position of their motor domain: kinesins with N-terminal (N-KIF), middle (M-KIF), and C-terminal (C-KIF) motor domain positions (Hirokawa et al., 2010; Yang et al., 2015; Kalantari and Filges, 2020).

To date, forty-five different KIF genes have been identified in the mouse and human genomes, including three M-Kifs (*Kif2a, Kif2b, and Kif2c*) and three C-KIFs (*Kifc1, Kifc2, and Kifc3*) (Miki et al., 2001; Hirokawa and Noda, 2008; Hirokawa et al., 2009; Kalantari and Filges, 2020).

N-KIF and C-KIF proteins share a similar structure comprising a motor domain, stalk region, and tail domain. The motor domain contains ATP and microtubule binding sites, enabling interaction with microtubules and movement along them through ATP hydrolysis. The tail domain, and in some cases the stalk region, mediates cargo recognition and binding. In M-Kifs, the motor domain is centrally located and does not function as a molecular motor but rather mediates microtubule depolymerization (Miki et al., 2001; Hirokawa and Noda, 2008; Hirokawa et al., 2010; Yang et al., 2015).

N-terminal KIFs typically move toward the plus-ends of microtubules, whereas C-terminal KIFs move toward the minus-ends. KIF2A and KIF2C are unique kinesins that depolymerize microtubules in an ATP-dependent manner (Hirokawa et al., 2009; 2010). Based on phylogenetic analysis, kinesins are classified into 16 families, from kinesin-1 to kinesin-14B (Miki et al., 2001; Kalantari and Filges, 2020).

Kinesins play a key role in intracellular transport in neurons, where they mediate anterograde axonal transport of various cargoes, such as vesicles and mitochondria, and specifically fast axonal transport (FAT) of proteins, lipids, and organelles, as well as transport of NMDA and AMPA receptors and mRNA in dendrites. Since component synthesis occurs in the neuronal cell body, anterograde transport is critical for neuronal growth, development, and functional communication. KIFs act as microtubule stabilizers (KIF26A and KIF21A) and depolymerizers (KIF2A and KIF2C), playing an important role in cell morphogenesis, which is particularly crucial for neurons. Kinesins also participate in mitosis, including assembly and function of the mitotic spindle, thereby regulating cell cycle dynamics (Merdes et al., 1996; Salina et al., 2002; Hirokawa and Noda, 2008; Raaijmakers et al., 2012; Niwa, 2015; Kalantari and Filges, 2020).

KIF defects are closely associated with various diseases, including spastic paraplegia, ALS, Alzheimer’s disease, Charcot-Marie-Tooth disease, multiple sclerosis, congenital fibrosis of the extraocular muscles type 1 (CFEOM1), and others (Kamal et al., 2001; Zhao et al., 2001; Reid et al., 2002; Hadano et al., 2006; Aulchenko et al., 2008; Yang et al., 2015).

### 2.3 Myosins

Motor proteins of the myosin superfamily represent the only known actin-based motor proteins to date. They utilize energy from ATP hydrolysis to generate mechanical force and directed movement along actin filaments. The wide range of cellular processes involving myosins arises from the unique structural, catalytic, and regulatory characteristics of different members of the myosin superfamily (Heissler and Sellers, 2016).

The structure of most myosins consists of a dimer comprising:

- A motor (catalytic) domain–a conserved “head” with actin and ATP binding sites that mediates movement;
- A neck region that regulates motor domain activity;
- A tail domain responsible for binding to cellular cargo and interacting with other proteins.

Analysis of the conserved motor domain divides the eukaryotic myosin superfamily into about 35 phylogenetic classes, including 12 expressed in humans. Based on their biochemical and mechanical properties, myosins are further grouped into four functional categories: fast motors, force-holding motors, strain sensors, and processive transporters (Heissler and Sellers, 2016).

The tail domains are the most variable regions of myosins. Unlike the conserved catalytic heads, tail domains differ between myosin classes and determine specific cellular functions through interactions with adaptor proteins. Recruitment of motor proteins to organelles or molecular complexes is typically mediated by binding of the tail domain to specific adaptors (Hartman and Spudich, 2012).

Myosins play a key role in a broad spectrum of cellular processes, including muscle contraction, cytokinesis, cell migration, vesicle transport, and signal transduction.

Defects in myosin function are closely associated with the development of various pathologies, including hereditary deafness, Griscelli syndrome, Usher syndrome, and other disorders (Hirokawa et al., 2010; Yang et al., 2015).

## 3 Role of motor proteins in stem cell proliferation

During embryonic development, the neural tube forms from the neural plate and is lined with pseudostratified neuroepithelium possessing stem cell-like properties. Neuroepithelial cells are polarized along the apical-basal axis, spanning the ventricular to pial surface. As neurogenesis progresses, the basal process of progenitor cells elongates, and they transform into radial glial progenitors (RGPs), which primarily undergo self-renewing asymmetric division, generating a radial glial cell and a neuroblast (Anthony et al., 2004; Dantas et al., 2016; Bertipaglia et al., 2018).

Defects in neuronal progenitor proliferation underlie brain developmental malformations such as primary microcephaly and megalencephaly, characterized by reduced and increased brain size, respectively. These pathologies can occur either in isolation or as part of more complex syndromes involving epilepsy, autism, intellectual disability, and cortical malformations. The pathogenesis of these disorders is caused by a broad spectrum of genetic defects, including, among others, mutations in genes encoding motor proteins, particularly kinesins (Pirozzi et al., 2018).

For example, pathogenic variants of the kinesin *KIF2A* in humans lead to microcephaly due to cell cycle delay in progenitor cells resulting from defects in ciliary disassembly, revealing a non-canonical role of KIF2A beyond its conventional transport function. Similarly, Kif2A knockout mice exhibit severe brain defects (Hirokawa et al., 2009; Kalantari and Filges, 2020).

Motor proteins in neurogenesis are not merely “cargo transporters.” They are active force generators and key components of mechanotransduction. In particular, they generate tension within the cytoskeleton, sense the mechanical properties of the environment, and convert mechanical signals into biochemical responses that determine the proliferation of neuronal progenitors.

During mitosis, motor proteins perform noncanonical, non-transport functions by contributing to spindle assembly and promoting chromosome congression to the metaphase plate. The forces required for chromosome movement toward the spindle poles are generated through motor-regulated microtubule dynamics: microtubule polymerization produces pushing forces, whereas microtubule depolymerization generates pulling forces (Do et al., 2026).

The main players in this process are dynein and kinesins, which generate opposing mechanical forces required for mitotic spindle assembly. Specifically, kinesin-5 (E.g.,5, KIF11) simultaneously binds to two antiparallel microtubules and drives their relative sliding, thereby increasing the distance between the spindle poles and causing them to separate (Valentine et al., 2006).

The NuMA–dynein complex generates an opposing pulling force, thereby maintaining balance. Interestingly, in addition to dynein, kinesin-14 also antagonizes kinesin-5 in this process. Like dynein, kinesin-14 moves toward the minus end of microtubules (Neahring et al., 2021).

### 3.1 Interkinetic nuclear migration

**FIGURE 2:**
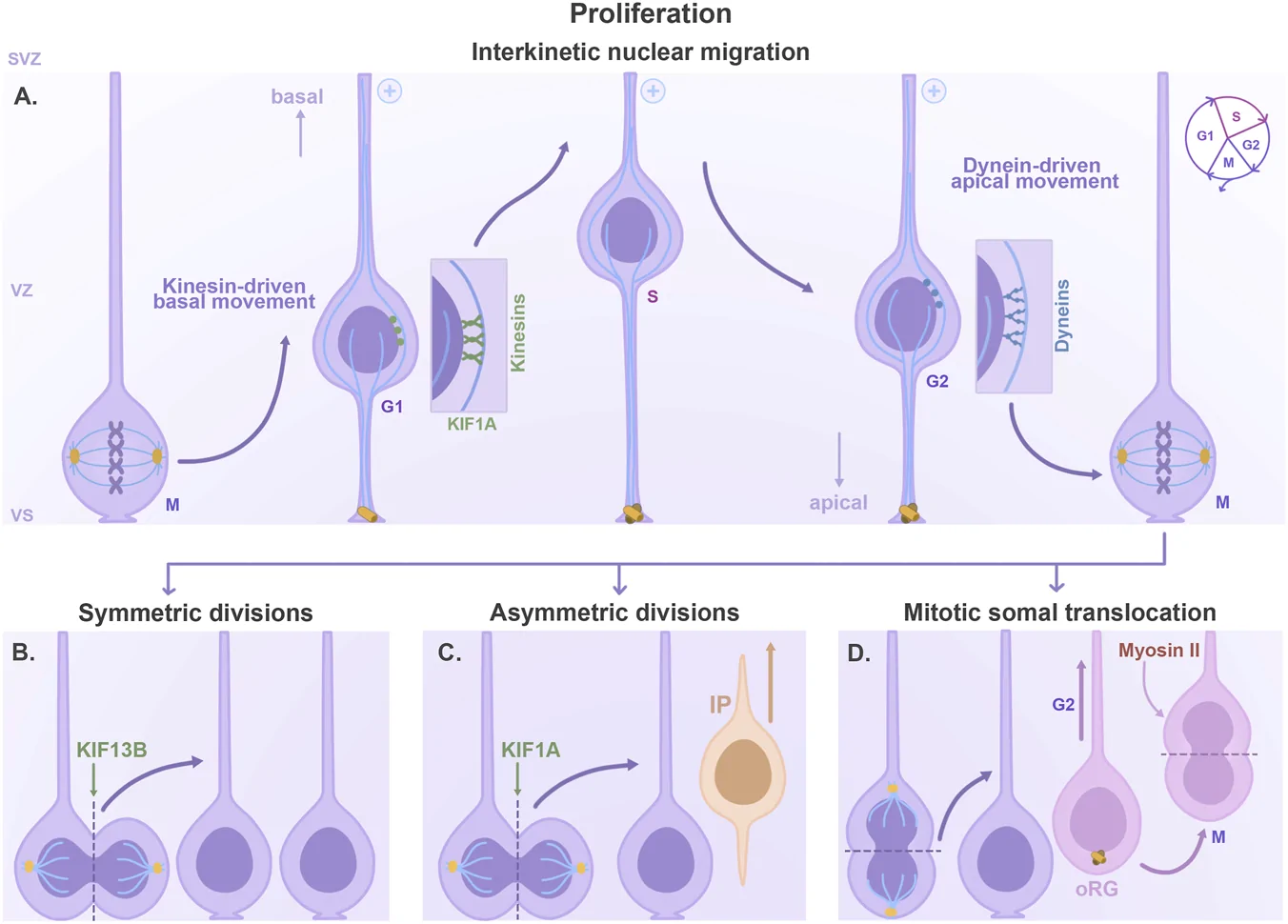
Roles of motor proteins in regulating neural progenitor proliferation. The schematic illustrates the involvement of motor proteins in the interkinetic nuclear migration, spindle orientation and positioning, cytokinesis, symmetric versus asymmetric cell divisions and mitotic somal translocation. By regulating nuclear dynamics and division plane orientation, motor proteins critically influence neural progenitor expansion and fate decisions during cortical development. **(A)** interkinetic nuclear migration; **(B)** symmetric divisions; **(C)** asymmetric divisions; **(D)** mitotic somal translocation. VS - ventricle surface; VZ - ventricular zone; SVZ - subventricular zone, IP - intermediate progenitor, oRG - outer radial glial progenitor.

Nuclei of both neuroepithelial and RGP cells exhibit cell cycle-dependent oscillatory movements known as interkinetic nuclear migration (INM) (Figure 2A) (Dantas et al., 2016; Bertipaglia et al., 2018).

Interkinetic nuclear migration is a process whereby, after completion of mitosis at the ventricular surface, the cell nucleus initiates basally directed movement during G1 phase, advancing toward the subventricular zone. There, the cell undergoes the S-phase. Subsequently, during G2 phase, the nucleus moves back in the apical direction–toward the neural tube lumen or, at later developmental stages, toward the ventricular surface, where the next mitotic division occurs. When the basal process of a radial glial progenitor (RGP) dramatically elongates, the amplitude of nuclear migration becomes restricted to the apical region of the cell within the ventricular zone (VZ) of the developing brain (Taverna and Huttner, 2010; Dantas et al., 2016; Bertipaglia et al., 2018).

Nuclear movement during interkinetic nuclear migration is driven by the transmission of mechanical forces through LINC (Linker of Nucleoskeleton and Cytoskeleton) complexes. These complexes are composed of SUN-domain proteins in the inner nuclear membrane and KASH-domain proteins (Nesprins) in the outer nuclear membrane, which in turn bind to components of the cytoskeleton (Rothballer and Kutay, 2013). The N-terminal region of SUN proteins interacts with the nuclear lamina in the nucleoplasm, whereas Nesprins in the cytoplasm bind to all major components of the cytoskeleton. Thus, SUN–KASH interactions mechanically couple the cytoskeletal and nucleoskeletal structures (Razafsky and Hodzic, 2015).

Apical nuclear movement is thought to involve dynein, whereas basal movement is mediated by kinesin. However, alternative models have also been proposed. Notably, during the development of the cerebral cortex, Nesprin-2 colocalizes and interacts with both dynein and kinesin-1. In both cases, the driving force is transmitted from the motor proteins to Nesprin-2 through a complex with BicD2 (Gonçalves et al., 2019; Zhou et al., 2024).

It is generally accepted that the primary driving force is generated by kinesin-mediated plus-end-directed movement along microtubules. Minus-end-directed motility driven by dynein serves a regulatory function, enabling cells to navigate around obstacles and finely tune the migration process (Meyerzon et al., 2009; Fridolfsson et al., 2010; Rothballer and Kutay, 2013).

#### 3.1.1 Basal nuclear migration during G1

In rat radial glial progenitors (RGPs), microtubule orientation indicates the involvement of a plus-end-directed motor protein, namely, kinesin, in mediating basal interkinetic nuclear migration (INM). Knockdown of the neuron-specific kinesin-3 family member *Kif1*a has been shown to selectively impair the basal phase of INM (Tsai et al., 2010; Dantas et al., 2016).

Pathogenic variants in *KIF1A* in humans cause KIF1A-associated neurological disorders (KAND) – a spectrum of severe CNS developmental malformations and neurodegenerative diseases, including progressive spastic paraplegias, microcephaly, encephalopathies, intellectual disability, autism, autonomic and peripheral neuropathy, optic nerve atrophy, as well as cerebral and cerebellar atrophy (Lin et al., 2025). Notably, patients with *KIF1A* mutations have been found to develop an unusual progressive form of microcephaly (Esmaeeli Nieh et al., 2015). Whether this effect is related to altered neurogenesis remains unknown. Although the kinesin Kif1a is known to pull the nucleus basally, its anchor in the nuclear envelope and the molecular pathway that recruits it in a G1-specific manner have yet to be identified (Bertipaglia et al., 2018).

#### 3.1.2 Apical nuclear migration during G2

Dynein motor activity drives nuclear translocation from the basal side toward the centrosome, which is anchored at the apical surface. In this context, dynein does not function as a conventional cargo transporter but as a force-generating motor driving apical nuclear migration (Tsai et al., 2007; Hu et al., 2013; Nakazawa and Kengaku, 2020).

The first direct evidence for motor protein involvement in INM was obtained through investigation of Lis1 function in the embryonic rat brain. In humans, mutations in *LIS1* lead to the development of classical lissencephaly, which may also be accompanied by agyria with thinning of the corpus callosum and epilepsy (Saillour et al., 2009).

Lis1 is a key regulator of cytoplasmic dynein, which moves toward the minus-end of microtubules. Experiments using *in utero* electroporation of shRNA against *Lis1* in the embryonic rat brain demonstrated that *Lis1* knockdown leads not only to nearly complete arrest of postmitotic neuronal migration, but also completely blocks INM in radial glial progenitors. Similar results were obtained with dynein heavy chain knockdown: apical nuclear migration in RGPs was completely blocked, whereas basal migration was partially preserved (Tsai et al., 2005; 2010; Bertipaglia et al., 2018).

In the zebrafish retina, mutations or altered expression of p150^Glued^, a component of the dynein regulatory complex dynactin, affect both basal and apical INM (Del Bene et al., 2008). In humans, mutations in DCTN1, the gene encoding p150^Glued^, result in pathologies such as distal spinal and bulbar muscular atrophy (dSBMA) and Perry syndrome (Levy et al., 2006; Ahmed et al., 2010; Yu et al., 2023).

Dynein regulators include, in addition to LIS1, its interactors Nde1 and Ndel1, as well as nuclear pore-associated proteins BicD2 and CENP-F, which participate in linking dynein to the nuclear envelope (Doobin et al., 2024).

Nde1 is a cytoplasmic dynein regulatory protein that contains multiple phosphorylation sites for the cell cycle-dependent protein kinase CDK1. In *Nde1* phosphomutants, both apical INM and mitosis in RGPs are blocked. Mutations in human *NDE1* cause severe forms of microcephaly or microlissencephaly, corpus callosum agenesis, brain developmental disorders, and psychiatric disorders including schizophrenia (Alkuraya et al., 2011; Bakircioglu et al., 2011; Paciorkowski et al., 2013; Kimura et al., 2015; Cabet et al., 2020). Mutations in *Ndel1* are embryonically lethal (Sasaki et al., 2005). NDE1 is essential for the progression of the cell cycle from G2 phase to mitosis (M phase). Its role in cell-cycle regulation likely contributes to the severe defects in neuronal proliferation observed in mouse embryonic fibroblasts (MEFs) isolated from E13.5 *Nde1* ^*−/−*^ mice, highlighting the critical importance of NDE1 in cerebral cortex neurogenesis (Alkuraya et al., 2011).

It has been established that dynein recruitment to the nuclear envelope occurs through two distinct pathways that act sequentially at different stages of G2 phase: the RanBP2–BicD2 pathway and the CENP-F pathway. In early G2, the nucleoporin RanBP2 (Nup358) binds the adaptor protein BicD2, which then recruits the dynein-dynactin-LIS1 complex to the nuclear envelope. In late G2, CENP-F becomes involved: it exits the nucleus and binds to Nup133 on the cytoplasmic side of the nuclear envelope. CENP-F then recruits the proteins Nde1 and Ndel1, which in turn recruit dynein and dynactin (McKenney et al., 2010; Splinter et al., 2010; Bolhy et al., 2011; Hu et al., 2013; Bertipaglia et al., 2018).

Mutations in human *BICD2* have been associated with cortical malformations such as polymicrogyria (PMG), spasticity, microcephaly, dominant spinal muscular atrophy, lissencephaly, and cerebellar hypoplasia (Ravenscroft et al., 2016; Huynh and Vale, 2017; Frasquet et al., 2020; Tsai et al., 2020; Abdel-Salam et al., 2022). Pathogenic variants in human *CENP-F* lead to hereditary ciliopathy with severe microcephaly–Stromme syndrome (Altieri et al., 2025), while defects in *NUP133* result in Galloway-Mowat syndrome (GAMOS), characterized by microcephaly and brain abnormalities (Fujita et al., 2018).

*BicD2* knockdown in rat embryonic brains caused nuclear arrest at a distance from the ventricular surface, whereas knockdown of *Nup133*, *CENP-F*, or *Nde1* resulted in nuclear arrest close to the ventricular surface. In both cases, radial glial progenitor (RGP) nuclei failed to enter mitosis, as reaching the apical ventricular surface is required for cell cycle progression (Hu et al., 2013; Doobin et al., 2016; Bertipaglia et al., 2018).

#### 3.1.3 Mitotic somal translocation

In primates, radial glial progenitors (RGPs) can give rise to a specialized cell type–outer radial glial cells (oRGCs), localized in the outer subventricular zone (oSVZ). The abundance of these cells varies across species: in primates, they form a substantial population, occupying an entire brain region–the oSVZ. Initially, oRGCs were considered unique to primates and were thought to underlie the development of cortical surface convolutions (gyrencephaly), whereas in rodents, which possess a smooth (lissencephalic) cortex, these cells were believed to be absent. However, subsequent studies have shown that oRGCs are present in rodents as well, although their numbers are extremely low (Hansen et al., 2010; Rash et al., 2019; Wang et al., 2011).

Unlike classical RGPs, oRGCs have lost contact with the ventricular surface. They possess a unipolar morphology and retain only a single basal process that can extend to the pial surface. Like RGPs, oRGCs are capable of self-renewal and differentiation into neurons. However, their distinguishing feature is a unique mechanism of nuclear movement–mitotic somal translocation (MST) (Figure 2D). Prior to division, the oRGC nucleus translocates outward along the basal process; notably, unlike interkinetic nuclear migration (INM) in RGPs, the centrosome remains continuously associated with the nucleus (Hansen et al., 2010; Ostrem et al., 2017).

It was shown that MST is largely regulated by myosin II: suppression of its regulatory enzyme ROCK completely blocked mitotic translocation, confirming the key role of this motor in the process. The precise mechanism by which myosin II organizes directed nuclear movement remains incompletely understood. One hypothesis suggests that it is related to its contractile activity, analogous to the role of myosin II behind the nucleus in migrating neurons (Wimmer et al., 2025).

The function of MST also remains a subject of debate. One hypothesis is that it is an adaptive mechanism associated with the increased size and structural complexity of the neocortical proliferative zones in primates, where a more flexible distribution of dividing cells is required (Dantas et al., 2016; Bertipaglia et al., 2018).

### 3.2 Symmetric and asymmetric divisions of RGPs

RGPs (aRGPs–apical radial glial progenitors) can divide either symmetrically (Figure 2B), expanding their pool, or asymmetrically (Figure 2C), generating either a postmitotic neuron, or basal radial glial progenitor (bRGP) or intermediate progenitor (IP), or a short neural precursor (SNP, also known as apical intermediate progenitors) (Noctor et al., 2004; Gal et al., 2006; Arai and Taverna, 2017).

With the exception of the latter, these cells migrate away from the ventricle for subsequent development. SNPs remain located in the VZ. Short neural progenitors were first described in the developing mouse neocortex, where they share several features with aRGPs, such as bipolar morphology, but possess a shorter basal process that is confined to the VZ. The apical and basal processes of SNP cells retract during mitotic division (Gal et al., 2006; Tan et al., 2016; Arai and Taverna, 2017).

Basal progenitors (bRGCs) still retain attachment to the basal (pial) surface via a basal process but lack an apical process. From an evolutionary perspective, the bRGC pool has substantially expanded in humans and other gyrencephalic species, resulting in the formation of two distinct basal germinal zones: the inner and outer subventricular zones (ISVZ and OSVZ, respectively) (Garcia-Moreno et al., 2012; Namba and Huttner, 2017).

Intermediate progenitors do not demonstrate INM and lack elongated processes, residing in the subventricular zone and ultimately dividing to generate two postmitotic neurons (Noctor et al., 2004; Bertipaglia et al., 2018).

During early development, most RGP divisions are symmetric, after which the percentage of asymmetric divisions gradually increases. Control of this balance must be carefully regulated to produce the correct numbers of neurons required for proper functioning of the cerebral cortex, on one hand, and, on the other hand, to maintain the pool of proliferating progenitors. The balance between symmetric and asymmetric RGP divisions is partially regulated by the actions of two kinesins, Kif1A and Kif13B, which play opposing roles in neurogenesis through their effects on the mitotic spindle during RGP division. Kif1A has been found to promote neurogenesis, whereas Kif13B promotes symmetric, non-neurogenic divisions. This finding correlates with changes in the angle between the RGP cell division axis and the ventricular surface (VS), indicating a role for these proteins in controlling mitotic spindle orientation (Helmer and Vallee, 2023). The involvement of the kinesin KIF20A in this process has also been demonstrated, with its activity promoting the transition from proliferative to differentiative divisions (Geng et al., 2018).

The inhibition of *Kif7*, whose role in this process is linked to cilia-dependent SHH signal regulation rather than intracellular transport, leads to impaired progenitor proliferation and neuronal differentiation. All patients carrying mutations in the *KIF7* gene exhibit developmental delay (DD) and intellectual disability (ID), associated with classic ciliopathy defects such as ventricular enlargement, hydrocephalus (up to hydrolethal syndrome), macrocephaly, corpus callosum agenesis (CC), and molar tooth sign (MTS). Some patients display anatomical cortical defects, including frontal lobe underdevelopment, brain atrophy, pachygyria, and heterotopia, which can manifest not only as DD and ID but also cause seizures in a quarter of patients (Dafinger et al., 2011; Ali et al., 2012; Barakeh et al., 2015; Pedraza et al., 2025).

*Kif7* −/− mice die at birth and are characterized by developmental malformations, including exencephaly, neural tube defects, microphthalmia, absence of olfactory bulbs, corpus callosum agenesis, and thinning of the dorsal cortex. Furthermore, in *Kif7* −/− embryos, TBR2 cells, which normally form a dense layer in the cortical SVZ, reached the brain surface, where they intermixed with postmitotic TBR1^+^ cells. Thus, intermediate progenitors and postmitotic neurons were no longer segregated, and no IZ was observed (Cheung et al., 2009; Pedraza et al., 2025).

Cytokinesis is the final stage of cell division, during which chromosomes and cytoplasmic contents are partitioned into two daughter cells. Abscission, the process by which the membrane connecting the two newly formed cells is severed, resulting in physical separation, completes cytokinesis. In epithelia-like tissues such as the brain, the orientation of the division plane can be critical for maintaining overall tissue architecture and daughter cell identity (Mierzwa and Gerlich, 2014; D’Avino et al., 2015; Johnson et al., 2017).

Cell division concludes with the formation of the midbody, a transient organelle that establishes the site of separation between the nascent daughter cells. During cytokinetic abscission, the final stage of cell division, one daughter cell inherits the midbody remnant, which it may then retain or expel. Studies of patients with microcephaly have revealed the involvement of midbody-associated proteins in pathogenesis, including mitotic molecular motors whose functions in this context are linked to mitotic regulation (Johnson et al., 2017).

At the molecular level, a key regulator of cytokinesis is the central spindlin complex, composed of the mitotic kinesin MKLP1 (KIF23) and the GTPase-activating protein MgcRacGAP (Zhao et al., 2006). Mutations in the human *KIF23* gene are associated with microcephaly (Naher et al., 2023), and knockdown in mice leads to premature neurogenesis and neuronal apoptosis, attributed to accelerated cell cycle exit, likely resulting from impaired mitotic spindle orientation and consequent cytokinesis defects (Naher et al., 2024).

Patients with variants in the *KIF14* gene exhibit intrauterine growth delay, severe microcephaly, brain and cerebellar hypoplasia, arhinencephaly, and agenesis of the occipital lobes and corpus callosum. The increased number of binucleated cells in tissue is consistent with the key role of KIF14 in mitosis as well as cytokinesis. These findings are supported by the detection of binucleated cells arising from cytokinesis failure in mammalian cells with *KIF14* knockdown (Carleton et al., 2006; Gruneberg et al., 2006; Fujikura et al., 2013; Kalantari and Filges, 2020).

Primary microcephaly can also be caused by pathogenic variants in genes encoding other mitotic kinesins, such as *KIF11* (as either an isolated or syndromic condition) and *KIF15* (as part of Braddock-Carey syndrome (BCS)) (Ostergaard et al., 2012; Sleiman et al., 2017).

A microcephaly-causing mutation in mice, called magoo, was found to reside in the vertebrate-specific kinesin *Kif20b* (a member of the kinesin-6 family related to Mklp1), which localizes to the midbody during cytokinesis. The magoo mutant cerebral cortex shows normal lamination, but upper-layer neurons are reduced due to increased apoptosis throughout neurogenesis. Midbody disruption, despite the absence of other cell cycle defects, appears sufficient to disrupt neurogenesis in the magoo cortex (Dwyer et al., 2011; Janisch et al., 2013; Johnson et al., 2017; McNeely and Dwyer, 2020).

The spatial positioning of the mitotic spindle is one of the key factors determining the type of cell division-whether symmetrical or asymmetrical. Spindle position is controlled by mechanical forces generated by motor proteins and transmitted to the cell’s cytoskeletal network.

Cortically anchored dynein, as part of the Gαi-LGN-NuMA-dynein complex, controls mitotic spindle orientation by interacting with astral microtubules and components of the actin cytoskeleton. This process is particularly important during asymmetric cell divisions (Lechler and Mapelli, 2021).

During mitosis, the mitotic spindle first assembles and captures chromosomes, and then, in metaphase, becomes centered within the cell, thereby defining the future plane of cytokinesis. The dynein–NuMA–LGN–Gαi complex localizes to the cell cortex directly above the spindle poles and determines spindle orientation. Dynein is thought to be the primary force generator acting on astral microtubules during spindle positioning: anchored at the mitotic cortex, it moves toward the minus ends of these microtubules, generating a pulling force on the spindle poles and thereby ensuring their correct positioning (Kiyomitsu, 2019; Lechler and Mapelli, 2021).

Kinesins play a supporting role in this process by regulating microtubule dynamics and stability. For example, members of the kinesin-6 family, MKLP1 (KIF23) and MKLP2 (KIF20A), play important roles during anaphase in the formation of the central spindle—a narrow region of antiparallel overlapping microtubules. Kinesin-13, in turn, depolymerizes microtubules, thereby regulating their dynamics and maintaining proper spindle architecture (Wu et al., 2019; Lechler and Mapelli, 2021; Poulos et al., 2022).

It should be noted that motor proteins not only generate and transmit mechanical signals within the cell but also act as a subject to mechanoregulation themselves. Feedback mechanisms exist whereby mechanical signals originating from the cellular microenvironment modulate the activity of motor proteins.

The dynein–NuMA–LGN–Gαi complex is associated with a feedback mechanism in which changes in membrane tension (including those dependent on the actomyosin complex) can influence the localization of the complex. Consequently, this regulates the orientation of the mitotic spindle, determining the division plane (whether it will be symmetrical or asymmetrical), and thus the cell fate (Fink et al., 2011; Finegan and Bergstralh, 2019; Tarannum et al., 2024).

Unlike dynein and kinesin, a direct influence of myosin on the processes of mitotic spindle orientation has not been demonstrated to date. However, there is evidence that myosin can influence the division plane by regulating cell shape through the actin cytoskeleton. Myosin activity is necessary for the formation of the actomyosin cortex and for cell rounding upon entry into mitosis. In the absence of mitotic rounding, cells cannot correctly interpret cortical signals that orient the mitotic spindle and divide along their long apical-basal axis (Chanet et al., 2017).

Mitotic rounding is driven by the reorganization of actin filaments into a uniform cortical layer, accompanied by a progressive increase in cortical tension. By the end of mitosis, a cortical tension gradient—from higher tension at the poles to lower tension at the equator—is established, driving cleavage furrow formation. Cytokinesis is executed through the accumulation of the actomyosin complex in the equatorial contractile ring, while the contractile cortex at the poles fine-tunes this process. This modulation is particularly important for regulating both symmetric and asymmetric cell divisions, such as those occurring in neuroblasts (Chugh and Paluch, 2018).

In addition to the activity of motor proteins and their associated complexes, the stability of mitotic spindle orientation is further supported by the viscoelastic properties of the cytoplasm, which resist spindle displacement and rotation (Xie et al., 2022).

Mechanical signals—including substrate stiffness, tensile and shear stress, and three-dimensional geometry—play a critical role in the asymmetric division of neural progenitors and their subsequent differentiation. These signals are sensed by specialized receptors and transduced through cytoskeletal structures, thereby regulating the proliferation, differentiation, migration, and self-renewal of neural stem cells (D’Angelo et al., 2011).

The perception of extracellular matrix (ECM) stiffness and topography is mediated by integrin-based adhesive contacts and the associated focal adhesion complexes. Their activation triggers signaling cascades, including the Rho GTPase and MAPK pathways, and modulates the activity of mechanosensitive ion channels. These events lead to the remodeling of nuclear architecture and to changes in the activity of mechanosensitive transcription factors, which ultimately direct cell differentiation toward the neuronal, oligodendrocytic, or astrocytic lineage (Pillai and Franze, 2024).

## 4 Role of motor proteins in neuronal polarization: multipolar stage and multipolar-to-bipolar transition

Postmitotic neurons generated from progenitors migrate to the subventricular zone (SVZ) and lower intermediate zone (IZ) of the neocortex. Upon reaching the lower IZ, neurons temporarily (for approximately 1 day) maintain a multipolar morphology. During this period, cells actively extend and retract numerous processes, interacting with environmental cues that guide their subsequent movement toward the cortical plate (Noctor et al., 2004; Tsai et al., 2005).

**FIGURE 3:**
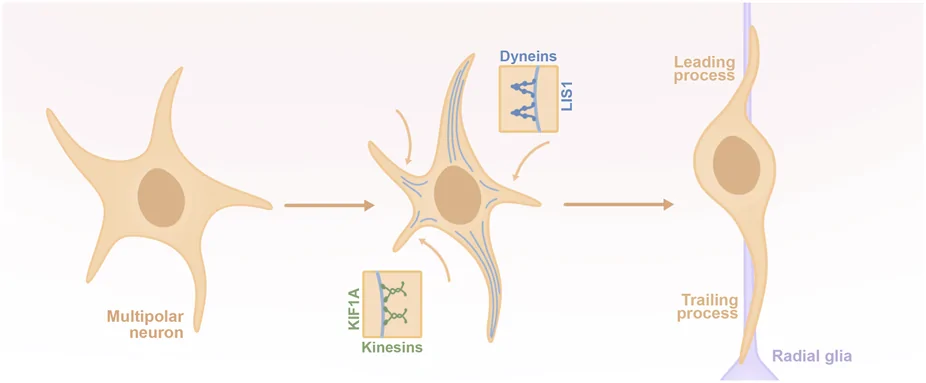
Motor proteins drive cytoskeletal remodeling during neuronal polarization. Schematic representation of the reorganization of the cytoskeleton and stabilization of processes by motor proteins required for the multipolar-to-bipolar transition during cortical development.

Under the influence of these signals, one process of the multipolar neuron continues to grow and gradually forms the axon. Another process thickens and becomes the leading process, while the remaining processes retract. As a result, the cell acquires a bipolar morphology and then begins radial migration, using radial glial cell fibers as guides (Figure 3) (Noctor et al., 2004; Tsai et al., 2007; Gavrish et al., 2024).

The molecular mechanisms triggering the neuronal transition from multipolar to bipolar state remain largely unclear. There is evidence that forces generated by cytoplasmic dynein and its regulator LIS1 play a key role in the formation and specification of the leading and axonal processes, and participate in axon elongation (Tsai et al., 2005; Yamada et al., 2010).

Inhibition of the cytoplasmic dynein pathway disrupts the multipolar-to-bipolar transition in neurons. Thus, inactivating *Lis1*, cytoplasmic dynein heavy chain, as well as *BicD2, CENP-F, Nup133, Nde1,* and *Ndel1* via RNA interference caused accumulation of multipolar cells in the subventricular zone of the embryonic rat cortex (Tsai et al., 2005; Hu et al., 2013; Doobin et al., 2016; Bertipaglia et al., 2018).

Similar effects were observed with the inhibition of the kinesin *Kif1a*, whose inactivation led to strong inhibition of the multipolar-to-bipolar transition and cell arrest at the multipolar stage. Notably, *Kif1a* inhibition in individual neurons caused non-cell-autonomous arrest of surrounding cells in the multipolar state, suggesting involvement of intercellular communication mechanisms. This effect may be explained by defective neurotrophin secretion, as Kif1a is involved in synaptic vesicle transport. Supporting this hypothesis, application of recombinant BDNF largely prevented the non-cell-autonomous effects of *Kif1a* inhibition; however, the persistence of the multipolar-to-bipolar transition arrest in directly transfected cells suggests that this phenotype cannot be explained solely by the transport-related functions of Kif1a. *Kif1a* inhibition additionally led to the initiation of expression of later neuronal differentiation markers, such as Tbr1, in multipolar cells, suggesting *Kif1a* involvement in gene expression control. Remarkably, some of these effects resembled those identified for doublecortin, the gene responsible for X-linked lissencephaly and a known *Kif1a* interactor (Bai et al., 2003; Carabalona et al., 2016; Bertipaglia et al., 2018).

Arrest of neurons at the multipolar stage prevents their subsequent radial migration along glial fibers and can lead to severe cortical developmental disorders, including periventricular heterotopia, subcortical band heterotopia, and lissencephaly (LoTurco and Bai, 2006; Falace et al., 2014; Carabalona et al., 2016).

Additionally, an autosomal-dominant missense mutation in human *KIF1A* has been described that disrupts neuronal migration processes and leads to frontal pachygyria (Carabalona et al., 2016).

Postmitotic neurons with mutations in the kinesin-6 *Kif20b* are unable to properly polarize and exhibit defects in dendritic and axonal branching. *In vitro*, mutant *Kif20b* neurons demonstrate severe polarization defects, and those neurons that do polarize have shorter axons with increased branching and longer minor neurites. These morphological changes are not associated with alterations in cell fate. Both axons and minor neurites of mutant neurons show increased width and longer growth cone filopodia, correlating with abnormal microtubule organization. *In vivo*, loss of *Kif20b* disrupted pyramidal neuron morphogenesis in the embryonic cortex. Mutant neurons had shorter and less branched apical dendrites that were incorrectly oriented relative to the cortical plane. It was confirmed that defects in neurite development were related not to impaired growth but to specification defects. Loss of *Kif20b* causes minor neurites to become longer (more axon-like), while axons become shorter and more branched (more dendrite-like) (McNeely et al., 2017).

## 5 Role of motor proteins in neuronal migration

### 5.1 Radial migration

Neuronal migration is another crucial step in the formation of functional neural circuits. Newly generated neurons must traverse a long distance from the germinal zone to the cortical plate, navigating through a space densely populated by other cells and packed with extracellular matrix. The greatest challenge is nuclear transport–the largest and most rigid part of the migrating neuron. Its translocation is mediated by pulling and pushing forces generated by cytoskeletal motors, whose activity is controlled by intracellular signals (Silva et al., 2019).

**FIGURE 4:**
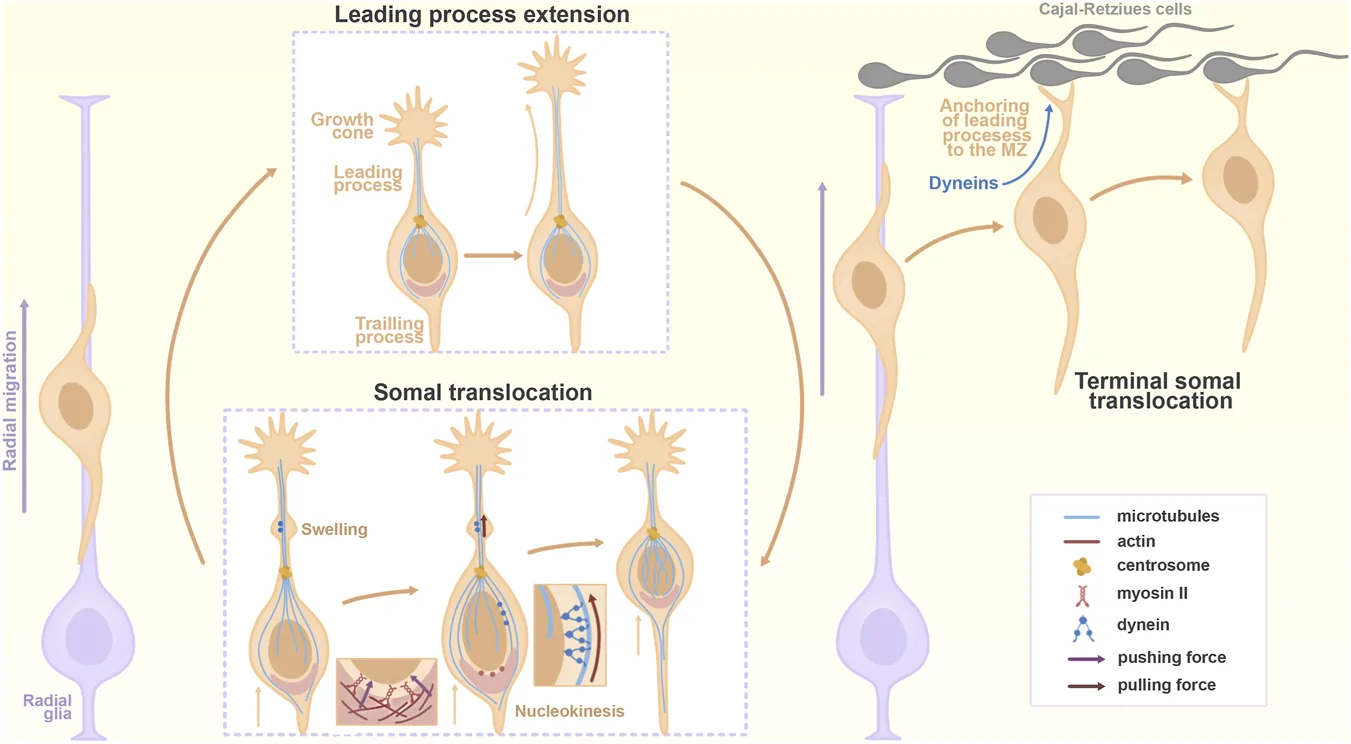
Roles of motor proteins in the regulation of radial neuronal migration. The schematic illustrates the roles of motor proteins in leading process extension, nucleokinesis, somal translocation and terminal somal translocation. Through regulation of microtubule and actomyosin dynamics motor proteins enable directed neuronal movement toward the cortical plate.

Neurons born at the ventricular surface in the cortex or in the SVZ migrate radially into the cortical plate, moving along the basal process of RGPs: the cell Soma glides along the RGP fiber through cycles of release and re-establishment of neuron-radial glia contacts. Migration continues until the leading process reaches the pial surface of the marginal zone, after which the Soma detaches from the RGP fibers and the nucleus translocates to its final position (Figure 4) (Cooper, 2013; Kawauchi, 2015).

Disruption of neuronal migration causes brain developmental malformations such as lissencephaly, characterized by an abnormally smooth brain with broad or absent gyri, cortical thickening with abnormal layer organization, and periventricular heterotopia, in which clusters of neurons fail to reach the cortical plate and are abnormally positioned along the lateral ventricular walls (Lu and Sheen, 2005; Hirota and Nakajima, 2017; Nakazawa and Kengaku, 2020; Tsai et al., 2020; Kattuoa and Das, 2025).

Migrating neurons exhibit pronounced polarity with three compartments: the growth cone of an axon, the leading process, and the cell body.

Cell migration is driven by self-generated adhesion forces. Migrating neurons have three distinct sites of adhesion generation that generate traction forces: one in the posterior process and two in the anterior process—one near the growth cone and one near the Soma (Jiang et al., 2015; Abuwarda and Pathak, 2020).

In this case, motor proteins are involved in the generation and transmission of mechanical forces. Cytoplasmic dynein, localized in the anterior process, exerts traction on microtubules extending from the centrosomal region. This ensures the translational movement of the microtubular cytoskeleton, including the centrosome (Vallee et al., 2009).

Myosin II is enriched in the leading process, where it induces contraction of the posterior process and pulls the centrosome and Soma forward during glial cell-driven migration (Solecki et al., 2009; Xu et al., 2019). Myosin II mediates nuclear migration by tightly regulating actomyosin contractions, which are cyclically generated behind the nucleus and push it forward (Javier-Torrent et al., 2021). Myosin II is activated by the RhoA–ROCK signaling pathway, enhancing actomyosin contractility. This leads to local tension in the actin cytoskeleton and the transmission of mechanical forces throughout the cell. These forces are then converted into cohesive forces through interactions between the cytoskeleton, adhesion complexes, and the extracellular matrix, thereby stimulating neuronal migration (Xu et al., 2019).

Migration typically occurs in two main phases: leading process extension and nuclear translocation (Figure 4) (Cooper, 2013).

At the first stage, polymerized F-actin in the growth cone pushes the plasma membrane forward. This process utilizes retrograde flow of actin filaments generated by myosin II, converting it into a driving force for growth cone expansion (Lowery and Vactor, 2009; Kerstein et al., 2015).

During neuronal migration, a swelling typically forms in the leading process, into which the centrosome and Golgi apparatus first move, followed by the nucleus sometime later. Nuclear movement occurs in a saltatory manner, likely reflecting mechanical resistance when transporting such a large and rigid structure through the considerably narrower channel of the leading process (Nishimura et al., 2017; Nakazawa and Kengaku, 2020).

This process requires coordinated action of cytoskeletal elements and motor proteins, particularly cytoplasmic dynein and its regulator LIS1, as well as non-muscle myosin II. Microtubules in migrating neurons are oriented such that their plus-ends point toward the nucleus, providing a connection between the centrosome and nuclear envelope through the LINC (Linker of Nucleoskeleton and Cytoskeleton) complex, formed by SUN (Sad1 and UNC-84) proteins on the inner nuclear membrane and KASH (Klarsicht, ANC-1, Syne Homology, also known as nesprins) proteins on the outer membrane. The main pulling force for movement is provided by cytoplasmic dynein, which, acting in complex with dynactin and the regulator LIS1, translocates the nucleus toward the centrosome through minus-end-directed motor activity. This is supported by experiments in the embryonic rat brain, where inhibition of cytoplasmic dynein or *LIS1* disrupted both centrosome movement and nuclear translocation (Tanaka et al., 2004; Tsai et al., 2007; Zhang et al., 2009; Sosa et al., 2013; Tapley and Starr, 2013).

Heterozygous mutations in *LIS1* are the most common cause of lissencephaly, while mutations in DCX cause X-linked lissencephaly (Toba and Hirotsune, 2012; Kattuoa and Das, 2025).

Migration defects were also observed in experiments involving inhibition of the dynein light chain *LIC1* expression, whose knockdown caused a sharp reduction in the number of neurons in the CP and accumulation of the majority of electroporated cells in the SVZ/IZ (Gonçalves et al., 2019).

Experiments using IUE-mediated knockdown have shown that loss of *BICD2* in cortical neurons disrupts corticogenesis by impairing radial migration of upper-layer excitatory neurons and formation of the classical mammalian “inside-out” cortex. Interestingly, deeper-layer TBR1^+^ neurons showed no significant defects in their radial migration. These findings suggest that migration of upper-layer neurons, which traverse greater distances during development, is more dependent on BICD2-mediated dynein activity than migration of deep-layer neurons (Will et al., 2019; Tsai et al., 2020).

Mutations in the actin-binding proteins nesprin-1/2 (*SYNE1*) lead to neuronal migration defects. In humans, mutations in nesprin-1 are associated with neurological disorders such as Meckel-Gruber syndrome, autosomal recessive cerebellar ataxia, bipolar disorder, autism, recurrent depression, visual impairments, early-onset neurodegenerative diseases, and cancer (Dawe et al., 2009; Synofzik et al., 2016; Fischer et al., 2022). Similarly, *Syne-1*−/−*Syne-2*−/− double knockout mice die perinatally, demonstrating severe defects in cortical organization (Zhang et al., 2007).

It has been shown that nesprin-2 can bind to both dynein and the dynein/dynactin complex component BICD2, as well as to the kinesin KIF5. One hypothesis about the role of nesprin-2 suggests that it may act as a molecular switch, regulating the activity of dynein and kinesin in response to spatiotemporal signals. Another possibility is that it facilitates the interaction between dynein and kinesin when both motors are active (Zhang et al., 2009; Olenick and Holzbaur, 2019).

Several hypotheses exist regarding the possible role of kinesin in processes where dynein is the dominant motor:

1. Braking function. Upon kinesin inhibition, nuclear translocation and neuronal migration accelerate, suggesting its possible function in restraining the forward movement of nuclei driven by dynein (Zhou and Kengaku, 2022).
2. Enhancing motility and flexibility. Several studies have shown that kinesin can induce dynamic nuclear rotation and transient backward displacements. These movements may help “disentangle” the nucleus from structural obstacles in the dense cytoplasm, facilitating passage through narrow spaces and promoting precise positioning (Wilson and Holzbaur, 2012).
3. Cargo anchoring. Kinesin may facilitate organelle attachment to microtubules, enhancing dynein-dependent transport. For example, the adaptor TRAK2 exhibits higher affinity for microtubules in the presence of kinesin, which increases mitochondrial transport efficiency (Fenton et al., 2021).

Although mice with single knockouts of either *Sun1* or *Sun2* genes do not display substantial brain abnormalities, *Sun1/2* double knockout mice exhibit reduced brain volume with severe lamination defects affecting the cerebral cortex, hippocampus, cerebellum, and olfactory bulb (Zhang et al., 2009).

Expression of a truncated nesprin-2 KASH domain in the embryonic rat brain dramatically slowed radial neuronal migration by nearly 90%, further emphasizing the importance of these proteins. It is also known that nesprin-2 can bind both kinesin and the dynein-dynactin complex, although the details of these interactions remain incompletely understood (Hu et al., 2013).

While it was previously believed that most perinuclear microtubules are oriented with their minus-ends toward the surface, recent studies have revealed mixed polarity. It has been shown that KIF5 (a plus-end-directed protein from the kinesin-1 family) participates in the nuclear translocation of migrating cerebellar granule cells (Wu et al., 2018). Pathogenic variants of *KIF5* in humans lead to spastic paraplegia type 10 (SPG10), Charcot-Marie-Tooth disease (CMT), amyotrophic lateral sclerosis (ALS), and neonatal intractable myoclonus (NEIMY) (Cozzi et al., 2025).

Inhibition of kinesin-5 (also designated, *E.g.,5* or *Kif11*) in migrating neurons results in faster but more erratic neuronal movement with a shorter leading process. Conversely, increased kinesin-5 expression both in culture and in the developing cerebral cortex leads to slowed migration and even its arrest. These data suggest that kinesin-5 regulates the speed and directionality of neuronal migration and possibly migration termination when the neuron reaches its destination (kinesin-5 is present in the neuron throughout the migration process, and its concentration peaks when the cell reaches its destination) (Falnikar et al., 2011).

Migration defects are also observed in neurons where the kinesin-4 family protein *KIF21B* is inhibited (Asselin et al., 2020).

In addition to microtubule motors, non-muscle myosin II, particularly myosin IIB, participates in nuclear movement. Suppression of its expression does not prevent centrosome movement but halts nuclear translocation, resulting in the centrosome positioning significantly ahead. Myosin II localizes to the rear of the nucleus, suggesting that it generates a pushing force, facilitating nuclear passage through the narrow constriction between the cell body and leading process. This mechanism likely operates in parallel with the pulling action of dynein along microtubules. However, other neuronal types, such as dissociated cerebellar granule cells, display a different scenario: myosin IIB localizes to the front of the nucleus immediately before its saltatory movement, indicating a possible pulling force exerted through the actomyosin complex (Bellion et al., 2005; Solecki et al., 2009; Martini and Valdeolmillos, 2010; Nakazawa and Kengaku, 2020).

In humans, myosin II defects cause pathologies including autism, schizophrenia, intellectual disability, and neurodegenerative diseases (Javier-Torrent and Saura, 2020), as well as microcephaly, cerebral and cerebellar atrophy, and deafness (Kasza et al., 2019). Likewise, genetic ablation of myosin IIb in mice leads to severe brain developmental abnormalities accompanied by pronounced neuronal migration defects. It has been shown that even a single point mutation in the motor domain of the myosin IIb isoform can disrupt neuronal migration in various neuronal subtypes. These findings have been further confirmed in experiments using selective myosin II inhibitors, particularly blebbistatin. These data emphasize that non-transport, force-generating functions of myosin II are essential for nuclear translocation and neuronal migration (Schaar and McConnell, 2005; Tsai et al., 2007; Dantas et al., 2016).

### 5.2 Terminal somal translocation

In the outer region of the cortical plate, radially migrating neurons undergo a final stage of migration independent of radial glia, called terminal somal translocation (TST) (Figure 4) (Nadarajah et al., 2001; Gonçalves et al., 2019).

This process involves detachment of the neuron from radial glia, contact of the leading process with the cortical marginal zone and its anchorage there, followed by rapid translocation of the cell Soma to its final position through shortening of the leading process (Gonçalves et al., 2019; Zhao et al., 2018).

This process also involves non-transport, force-generating functions of molecular motors, in particular dynein. Knockdown of the dynein light chain *LIC2* caused a sharp decrease in the number of cells reaching the uppermost layers of the CP. These cells had elongated apical dendrites contacting the marginal zone, but their cell bodies were located in deeper cortical layers (Gonçalves et al., 2019).

## 6 Role of motor proteins in neuritogenesis

### 6.1 Axonogenesis. Axonal growth cone, axonal navigation, formation of the corpus callosum

Neurons transmit information in the nervous system using two types of processes: axons, specialized for rapid long-distance information transmission, and dendrites, which receive and process data from multiple sources. These functions are enabled in part by the specific organization of microtubules (MTs) within the processes. In mammalian axons, most microtubules are oriented with their minus-ends toward the cell Soma and plus-ends toward the axon periphery, forming a parallel array, whereas in dendrites their orientation is mixed. In dendrites, dynamic tyrosinated microtubules are oriented with minus-ends toward the cell Soma, while more stable, acetylated microtubules are oriented in the opposite direction. These processes of microtubule organization represent well-established non-transport functions of motor proteins in regulating cytoskeletal polarity and architecture (Van De Willige et al., 2016; Tempes et al., 2020).

The growth of an axon or dendrite can be thought of as the interaction of three mechanical systems: the actin apparatus, which ensures protrusion and retrograde actin flow; the microtubule apparatus, which ensures movement and navigation; and adhesion receptors, which link the cytoskeleton to the substrate (Vitriol and Zheng, 2012).

The mechanisms organizing MTs in growing neurites are largely analogous to those organizing mitotic spindle microtubules during mitosis, suggesting the involvement of similar molecular motors (Sharp et al., 2000).

In early stages of neurite outgrowth, microtubule sliding relative to each other, mediated by kinesin-1 (KIF5), breaks the spherical symmetry of undifferentiated neurons and initiates protrusion formation. KIF5 forms a crossbridge between adjacent microtubules, with the microtubule bound to the heavy chain C-terminus becoming “cargo” for kinesin-1, while the other microtubule serves as a track. Microtubule minus-ends push against the plasma membrane at process tips, promoting their extension. *In vivo* experiments in *Drosophila* have shown that a *KIF5* mutation that reduces its microtubule-sliding ability without affecting its cargo transport function causes severe defects in neurite outgrowth as well as axonal and dendritic patterning (Winding et al., 2016; Lu and Gelfand, 2017). At a later stage, dynein is involved, pushing minus-end microtubules out of the neurites, drawing in plus-end microtubules, and pushing them toward the tips of the process, continuing its elongation (Oelz et al., 2018).

As neurites continue to grow, the growth cone engages its cytoskeleton for forward movement and rotation. This occurs in three stages, each influenced by environmental factors: protrusion, engorgement, and consolidation. Growth cone motility depends on the dynamics of the actomyosin complex. Myosin generates contractile forces in the actin network, controlling the retrograde flow of F-actin in the growth cone. This F-actin flow, in turn, responds to adhesion signals sensed by membrane receptors, determining the directional movements of the growth cone (Lowery and Vactor, 2009).

**FIGURE 5:**
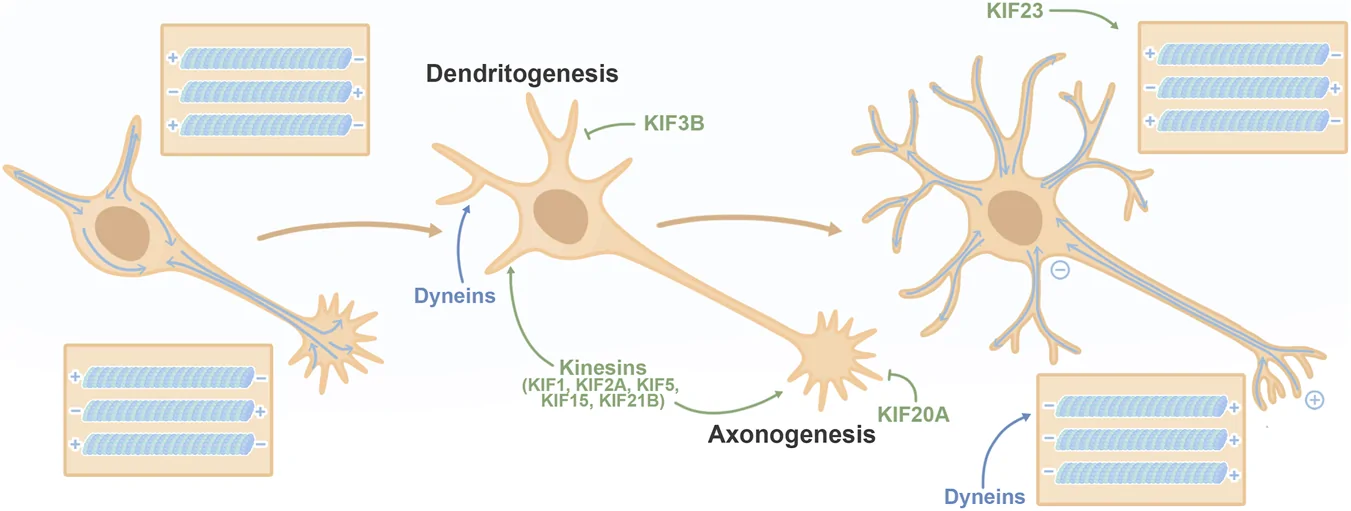
Motor proteins roles during neurite outgrowth. The schematic illustrates how motor proteins are involved in the formation of axons and dendrites. In developing axons microtubules progressively adopt a uniformly oriented microtubule array (plus-end-out), supporting directed kinesin- and dynein-mediated transport. Whereas dendrites maintain a mixed microtubule polarity, resulting in distinct transport dynamics that shapes the growth and identity of dendrites.

It has been shown that initially all neurites contain microtubules of mixed polarity; at later stages, axonal orientation changes to uniform (plus-ends out), while mixed orientation persists in dendrites (Figure 5). This process is mediated by microtubule sorting in the growing axon, driven by cytoplasmic dynein (Ahmad et al., 2000; Roossien et al., 2014; Lu and Gelfand, 2017).

Microtubules oriented with minus-ends toward the cell body are transported along the actin framework into growing processes via a “sliding filament” mechanism, while microtubules with minus-ends outward are removed from the axon (Sharp et al., 2000; Lu and Gelfand, 2017).

The sliding filament model proposes that a portion of dynein in the axon is immobilized (through interaction with stable microtubules and/or actin), while the dynein motor domain interacts with short microtubule polymers, advancing microtubules with distally positioned plus-ends down the axon as the motor domain moves toward the microtubule minus-end. In addition to transporting microtubules, dynein functions as a “gatekeeper,” moving microtubules with distally positioned minus-ends toward the Soma, excluding them from the axon. Neurons lacking functional dynein will still transport microtubules, likely via kinesin-3, but now microtubules with distal minus-ends will also enter the axon (Zheng et al., 2008).

It was shown that dynein inhibition through dynactin disruption halts microtubule translocation into growing neurites (Ahmad et al., 1998; Sharp et al., 2000).

Microtubule sliding is active only in growing neurons and stops as they mature. The “mitotic” kinesin-6 *KIF20A (MKLP1)* has been shown to be responsible for inhibiting this process. Its overexpression in young neurons causes axon growth arrest (Lu et al., 2013; Del Castillo et al., 2015).

Depletion or pharmacological inhibition of the kinesin *KIF1C* in embryonic neurons results in shorter and less branched axons. Axonal growth cones are characterized by a smaller size and fewer filopodia occupied by microtubules (Muralidharan and Baas, 2019; Joseph et al., 2021).

Knockdown of kinesin *KIF15* (kinesin-12) significantly increases the rate of axon growth but reduces their branching, and also leads to the formation of longer and thinner dendrites. This phenotype may be due to dynein-induced enhancement of plus-end MT distribution into dendrites, as KIF15 prevents such MTs from entering dendrites (Lin et al., 2012; Xu et al., 2014; Joseph et al., 2021).

Similar to *KIF15, KIF2A* also has a similar axonal phenotype. The M-kinesin KIF2A is proposed to depolymerize MTs at the axonal growth cone edge and suppress collateral branch growth. *KIF2A* knockout causes increased MT stability by preventing depolymerization and degradation. This in turn causes cortical developmental abnormalities including agyria and pachygyria, subcortical band heterotopia, and corpus callosum anomalies (Maor-Nof et al., 2013; Gilet et al., 2020; Joseph et al., 2021; Ruiz-Reig et al., 2023).

The corpus callosum (CC) is a bundle of axons that interconnects the cerebral hemispheres and conducts neural transmission of signals associated with motor, sensory, and cognitive functions. After the neuron reaches its final position in the cortical plate, corpus callosum axons project toward the midline as a compact bundle, cross into the contralateral hemisphere, where they form synapses (Gavrish et al., 2024; Lanzarone et al., 2025).

Corpus callosum abnormalities are among the most common central nervous system developmental malformations detected by prenatal ultrasound, occurring in 2%–6% of children with neurodevelopmental abnormalities, and can either be found in isolation or as part of a complex syndrome. CC developmental pathologies lead to delayed motor and cognitive functions, epilepsy, impaired social and speech development, autism, attention deficit disorder, and schizophrenia (Lanzarone et al., 2025).

KIF21B is a kinesin that facilitates intracellular transport and controls microtubule dynamics. Patients with mutations in the *KIF21B* gene exhibit brain developmental malformations, including agenesis of the corpus callosum (ACC) and microcephaly, developmental delay, and intellectual disability. Homozygous *KIF21B* knockout (KO) mice display severe morphological abnormalities, including microcephaly and partial loss of commissural fibers, cognitive deficits, and reduced dendritic complexity. *In vitro*, *KIF21B* ^−/−^ cultured neurons show less branched dendrites with lower spine density and shorter axons (Asselin et al., 2020; Tempes et al., 2020; Narayanan et al., 2022).

In neurons, KIF21B is present in both axons and dendrites and is particularly abundant in growth cones, where it regulates microtubule dynamics by promoting or terminating their growth through accumulation at MT plus-ends. Notably, experimental data indicate that corpus callosum agenesis upon *KIF21B* inhibition is directly related to defects in axon growth and branching rather than to axonal guidance defects (Guo et al., 2015; Lipka et al., 2016; Muhia et al., 2016; Asselin et al., 2020).

Inhibition of *KIF11* accelerates axonal growth by increasing microtubule mobility in the axon shaft and promoting microtubule invasion into the growth cone. It also disrupts growth cone guidance by preventing proper turning (Kahn et al., 2015).

Defects in kinesin *KIF7* cause several syndromes characterized by multiple developmental malformations, including corpus callosum agenesis, namely, acrocallosal syndrome, Joubert syndrome, and Al-Gazali-Bakalinova syndrome (Al-Gazali and Bakalinova, 1998; Dafinger et al., 2011; Walsh et al., 2013; Barakeh et al., 2015). Knockdown of *KIF7* with gene-specific *KIF7* shRNA electroporated into the developing mice cerebral cortex at E14.5 resulted in a marked decrease in axons crossing the midline toward the contralateral cortex, along with diminished branching and overall length of apical neurites (Guo et al., 2015).

### 6.2 Dendritogenesis

The formation of nervous system architecture during development requires precise regulation of dendritogenesis: individual neurites may elongate, branch, retract, or shorten. Various abnormalities in dendritic structure and dendritic spine morphology are commonly associated with cognitive impairments and intellectual disability. *Postmortem* studies of neural tissue have demonstrated altered dendritic tree architecture in the hippocampus, dentate gyrus and cerebral cortex of affected patients with neurodevelopmental disorders (Quach et al., 2021).

Unlike axons, dendrites must organize not only microtubules oriented with their minus ends toward the cell body during their growth, but also those oriented with their plus ends toward the Soma, a process that requires force generation driven by the activity of molecular motors. CHO1/MKLP1, also known as KIF23, has been shown to mediate this type of antiparallel MT sliding *in vitro*. *KIF23* knockdown in primary neurons impairs dendritic growth, while axons develop normally. Those dendrites that do develop have thinner processes, acquiring a more axon-like phenotype (Nislow et al., 1992; Sharp et al., 1997; Joseph et al., 2021).

*KIF3B* knockdown in developing neuronal cultures has been found to result in short or completely absent neurites. *KIF3B* knockdown in dissociated cortical neurons caused an overall increase in dendritic spine density. Collectively, recent studies suggest that the role of KIF3B is to inhibit dendritic growth and dendritic spine formation. *KIF3B* defects cause retinal atrophy, schizophrenia, and autosomal dominant ciliopathy (Alsabban et al., 2020; Cogné et al., 2020; Adams et al., 2024).

*KIF11* knockdown in postmitotic neurons leads to induction of branching in both axons and dendrites, significant axon elongation, and increased overall spine density. The phenotype appears to be partially due to reduced propensity of processes to undergo retraction (Kahn et al., 2015; Swarnkar et al., 2018).

In dendrites, *KIF11* inhibition alters dendritic morphology and microtubule organization. Transient inhibition enhances microtubule dynamics and dendritic growth, whereas transient activation suppresses both processes. Under *KIF11* inhibition, dendrites become shorter and thinner and contain a higher proportion of minus-end-out microtubules, suggesting that kinesin-5 normally restricts the transport of these microtubules into dendrites (Kahn et al., 2015; Wingfield et al., 2026).

Interestingly, KIF11 regulates dendritic branching differently during neuronal development: deletion of *KIF11* in mature neurons increases dendritic branching, whereas in immature neurons loss of *KIF11* reduces branching. Mutations in *KIF11* cause the rare disorder microcephaly with or without chorioretinopathy, lymphedema, or intellectual disability (MCLID). In rodent models, homozygous *Kif11* knockout mice die before the blastocyst stage because *Kif11* dysfunction induces monopolar spindles and polyploidy, while heterozygous mice develop normally. Patients with MCLID are also heterozygous. Together, these findings identify KIF11 as a key regulator of microtubule stability and dendritic architecture in mature neurons, with MCLID pathogenesis partly linked to microtubule destabilization and reduced dendritic complexity (Peng et al., 2025; Wingfield et al., 2026).

The distinct microtubule organization in axons and dendrites dictates different transport rules in these compartments (Figure 5). In mammalian neurons, due to mixed microtubule orientation, both dynein and kinesins are capable of directing cargo into dendrites (Tempes et al., 2020). During neuritogenesis, motor proteins also function as regulators of cytoskeletal architecture by organizing and stabilizing microtubule arrays within growing neurites, thereby contributing to neurite specification and the establishment of neuronal polarity independently of their cargo transport activity.

Defects in the dynein-dynactin complex formation led to impairments in dendritogenesis. Specifically, mutations in *Dlic2*, the gene encoding dynein intermediate chain, result in shortened dendritic branches, a shift in the ratio between distal and proximal dendrites toward proximal, reduced total branching area, and axon thickening, as shown in *Drosophila* experiments (Singhania and Grueber, 2014). A similar phenotype was observed with defects in the kinesin heavy chain gene (*khc*) (Satoh et al., 2008). Manipulations of the level or activity of other dynein intermediate and light chains lead to substantial dendrite shortening or reduced complexity of dendritic arbors *in vitro* (Zhou et al., 2012; Islam et al., 2015; Tempes et al., 2020). However, studies in mice showed that dendrites of homozygous mutants with defects in the dynein light intermediate chain gene *Dync1li1* conversely became longer and extended farther from the Soma than dendrites of wild-type animals, though dendritic tree complexity did not change statistically (Banks et al., 2011).

The important role of dynein in dendritogenesis is further confirmed by studies of dynactin, dynein activators, and other regulatory proteins. Loss of the dynein activity regulator Lis1 leads to reduced dendrite number in *Drosophila* mushroom body neurons. *p150* ^*Glued*^ knockdown resulted in substantial simplification of rat hippocampal neuron dendritic trees *in vitro*. *Drosophila* studies showed that mutation of the *Bicd* gene (homolog of human *BICD1* and *BICD2*) produces a similar phenotype, whereas its overexpression stimulates dendritic branching and growth (Bianco et al., 2010; Tempes et al., 2020). These data are further supported by studies in *C. elegans* (Aguirre-Chen et al., 2011).

Loss of the dynein cofactor *NudE* leads to abnormal dendritic branching, also affecting Golgi apparatus compartments located in dendrites. Neurons lacking NudE exhibit increased microtubule dynamics, which is likely contributes to abnormal dendritic growth and branching. These dendritogenesis defects are rescued by increasing levels of *Lis1*, another dynein cofactor that interacts with NudE in a three-component complex (Arthur et al., 2015).

The role of kinesins in dendritogenesis was demonstrated using the kinesin-1 family, particularly *KIF5*. Suppression of its expression in developing hippocampal neurons led to a substantial reduction in dendritic complexity (Geiger et al., 2014).

Patients with pathogenic variants of *KIF1B* exhibit Charcot-Marie-Tooth disease type 2A (Zhao et al., 2001), while defects in *KIF1C* cause spastic ataxia (Mahale et al., 2024; Granath et al., 2025), hypomyelination, tremor and dystonia (Marchionni et al., 2019), as well as hereditary spastic paraplegia and cerebellar dysfunction, however molecular mechanisms underlying these disorders yet to be fully characterized (Dor et al., 2014; Siddiqui et al., 2022). Knockdown of kinesin-3 family members (KIF1A, KIF1B, and KIF1C) also leads, in addition to impaired intracellular transport, to simplification of dendritic trees, suggesting that future studies should address whether these phenotypes may also involve non-transport functions of these proteins (Lipka et al., 2016; Tempes et al., 2020).

Members of the kinesin-13 family have a catalytic domain in the center of the protein and do not move along microtubules, but rather use their ATPase activity for microtubule depolymerization (Vicente and Wordeman, 2015). In the nervous system, *KIF2A* is the only kinesin-13 family member expressed in postmitotic neurons. *KIF2A* missense mutations in patients lead to cortical gyrification abnormalities and microcephaly, and also cause drug-resistant epilepsy and intellectual disability (Gilet et al., 2020). *KIF2A* knockout in dentate gyrus neurons leads to aberrant morphological changes in axons, cell bodies, and dendritic spines in mice, as well as the formation of cells with multiple axonal neurites *in vitro*, with excessively elongated dendrites displaying axonal (Tau1-positive) rather than dendritic (MAP2-positive) characteristics. These results suggest that *KIF2A* loss may disrupt neurite differentiation and cause the development of multiple short axons in the hippocampus, leading to the formation of complex networks in the dentate gyrus (Homma et al., 2018). It has also been shown that *Kif2a* gene knockout in cultured neurons significantly increases not only the number of axons but also the number of primary dendrites, while *Kif2a* knockout hippocampal primary neurons display shorter primary and secondary dendrites (Gilet et al., 2020).

## 7 Role of motor proteins in synaptogenesis

Synaptogenesis is a final phase in the formation of neural networks and overall brain connectivity architecture. Genes involved in synaptogenesis are frequently associated with neurological and psychiatric disorders. Previously described processes, such as neuronal differentiation, migration, axon guidance, and dendrite morphogenesis, can influence synaptogenesis outcomes (Estrin and Bhavnani, 2020).

However, according to current understanding of synaptogenesis processes, the contribution of motor proteins is directly related to their transport functions in delivering specific cargoes to forming synaptic terminals. The molecular mechanisms of synaptogenesis are described in detail in the review by Qi et al. (2022).

## 8 Concluding remarks and future perspectives

Cytoskeletal motor proteins play a dual role in cortical development. In addition to mediating intracellular transport, dyneins, kinesins, and myosins, as well as their interactors, serve as critical regulators of neuronal proliferation, migration, and differentiation through their non-transport functions. Motor proteins are involved in the generation, transmission, and perception of mechanical forces within the cell. They regulate cytoskeletal tension, actomyosin contractility, and pulling and traction forces, which significantly influences neurogenesis. Non-transport functions directly link the molecular activity of motor proteins to phenotypic manifestations of cortical developmental disorders such as microcephaly, lissencephaly, corpus callosum agenesis, and psychiatric disorders. Recent studies have only begun to unveil this complex network of corticogenesis regulation.

For example, one of the most studied genes is *KIF23*. This gene is known to be highly expressed in proliferating neural stem cells, and its pathogenic variants have been clinically associated with microcephaly (Karaca et al., 2015; Polioudakis et al., 2019). According to recent studies, in this case, microcephaly arises from premature cell cycle exit of neural progenitors followed by apoptosis. This is linked to the fact that loss of *KIF23* leads to impaired mitotic spindle orientation and an increase in asymmetric cell divisions, resulting in a reduced pool of proliferating progenitors and consequently an insufficient number of neurons required for normal brain development (Naher et al. 2023; Naher et al. 2024).

We have summarized the available data on the major motor proteins and their encoding genes involved in neurogenesis and systematized them in Table 1. For each protein, we present its non-transport functions at key stages of nervous system development, including proliferation of neural progenitors, polarization and migration, as well as axonogenesis and dendritogenesis. For each gene, we also indicate human neurodevelopmental disorders associated with pathogenic variants.

**TABLE 1:** Non-transport functions of motor proteins in corticogenesis and related neurodevelopmental disorders.

Family	Protein/gene	Phenotype in patients	Role in proliferation	Role in polarization	Role in migration	Role in neuritogenesis
Dynein and its cofactors	Dynein	Amyotrophic lateral sclerosis (ALS), lissencephaly, Alzheimer’s disease (Sharp et al., 2000; Yang et al., 2015), cerebral cortex malformations, congenital muscular dystrophy (CMD), Charcot-Marie-Tooth disease (CMT), microcephaly, dysgyria, peripheral neuropathies, SMA-LED (Marzo et al., 2019; Romero et al., 2023)	Interkinetic nuclear migration (INM) (apical) (Tsai et al., 2007; Hu et al., 2013; Nakazawa and Kengaku, 2020)	Formation and specification of the leading and axonal processes, multipolar-bipolar transition (Tsai et al., 2005; Hu et al., 2013; Doobin et al., 2016; Bertipaglia et al., 2018)	Pulls the nucleus forward during neuronal migration and is involved in terminal somal translocation (Tanaka et al., 2004; Tsai et al., 2007; Zhang et al., 2009; Sosa et al., 2013; Tapley and Starr, 2013; Gonçalves et al., 2019)	Sorts microtubules in the growing axon, affects the growth and branching of dendrites, and the complexity of dendritic trees (Ahmad et al., 2000; Roossien et al., 2014; Lu and Gelfand, 2017; Sharp et al., 2000; Tempes et al., 2020)
	Dynactin (p150Glued/D*CTN1*)	Distal spinal bulbar muscular atrophy (dSBMA) and Perry syndrome (Ahmed et al., 2010; Levy et al., 2006; Yu et al., 2023)	Participates in both apical and basal nuclear migration (Del Bene et al., 2008; Dantas et al., 2016)	​	​	Stimulates dendritic branching and growth (Singhania and Grueber, 2014; Tempes et al., 2020)
	Dynactin (p50, dynamitin/*DCTN2*)	-	​	​	​	Inhibits dendritic branching (Zhou et al., 2012; Islam et al., 2015; Tempes et al., 2020)
	*LIS1*	Lissencephaly, agyria with thinning of the corpus callosum and epilepsy (Ortug et al., 2023; Saillour et al., 2009)	Essential for INM (Tsai et al., 2005 ; 2010; Bertipaglia et al., 2018)	Multipolar-bipolar transition (Tsai et al., 2005; Bertipaglia et al., 2018)	Involved in dynein-mediated nuclear movement (Tanaka et al., 2004; Tsai et al., 2007; Zhang et al., 2009; Sosa et al., 2013; Tapley and Starr, 2013)	Affects the correct branching of shoots (Arthur et al., 2015)
	*NDE1*	Severe microcephaly, agenesis of the corpus callosum, fetal brain malformation, microlissencephaly and psychiatric disorders, including schizophrenia (Bradshaw, 2016; Cabet et al., 2020; Paciorkowski et al., 2013)	Apical INM, mitosis in radial glial progenitors (RGPs) (Alkuraya et al., 2011; Bakircioglu et al., 2011; Paciorkowski et al., 2013; Kimura et al., 2015; Cabet et al., 2020)	Multipolar-bipolar transition (Doobin et al., 2016; Bertipaglia et al., 2018)	​	​
​	*BICD2*	Polymicrogyria, spasticity, microcephaly, dominant spinal muscular atrophy, lissencephaly, and cerebellar hypoplasia (Abdel-Salam et al., 2022; Frasquet et al., 2020; Huynh and Vale, 2017; Ravenscroft et al., 2016; Tsai et al., 2020; Will et al., 2019)	Apical INM (Bertipaglia et al., 2018; Hu et al., 2013)	Multipolar-bipolar transition (Hu et al., 2013; Doobin et al., 2016; Bertipaglia et al., 2018)	Radial migration of neurons in the upper layers, forming the cortex “from the inside out” (Tsai et al., 2020; Will et al., 2019)	Stimulates branching and dendritic growth (Bianco et al., 2010; Tempes et al., 2020)
	*CENP-F*	Primary hereditary microcephaly, Stromme syndrome (ciliopathy with severe microcephaly) (Altieri et al., 2025)	​	Multipolar-bipolar transition (Bolhy S. et al., 2011; Doobin et al., 2016; Bertipaglia et al., 2018)	​	​
	*NUP133*	Galloway-Mowat syndrome (GAMOS) (Fujita et al., 2018)	​	Multipolar-bipolar transition (Hu et al., 2013; Doobin et al., 2016; Bertipaglia et al., 2018)	​	​
Kinesins	*KIF1A*	KIF1A-associated neurological disorders (KAND) (Dantas et al., 2016; Rao et al., 2025)	Pulls the nucleus basally in INM Promotes asymmetric neurogenic divisions of the RGP (Bertipaglia et al., 2018; Dantas et al., 2016; Helmer and Vallee, 2023; Rao et al., 2025)	Multipolar-bipolar transition (Bai et al., 2003; Carabalona et al., 2016; Bertipaglia et al., 2018)	​	Affects dendritic branching (Lipka et al., 2016; Tempes et al., 2020)
	*KIF1B*	Charcot-Marie-Tooth disease type 2A (Zhao et al., 2001)	​	​	​	Affects dendritic branching (Lipka et al., 2016; Tempes et al., 2020)
	*KIF1C*	Spastic ataxia, hypomyelination, tremor, dystonia, hereditary spastic paraplegia and cerebellar dysfunction (Dor et al., 2014; Granath et al., 2025; Mahale et al., 2024; Marchionni et al., 2019; Siddiqui et al., 2022)	​	​	​	Affects axon growth and branching, the movement of microtubules into the filopodia of the axon growth cone, and the branching of dendritic trees (Joseph et al., 2021; Lipka et al., 2016; Tempes et al., 2020)
	*KIF2A*	Microcephaly, agyria, pachygyria, heterotopia of the basal cords, and corpus callosum anomalies. (Joseph et al., 2021; Kalantari and Filges, 2020)	Affects the cell cycle by participating in the disassembly of cilia (Hirokawa et al., 2009; Kalantari and Filges, 2020)	​	​	Depolymerizes MT at the edge of the axon growth cone and inhibits branching Affects the specification of axons and dendrites (Maor-Nof et al., 2013; Gilet et al., 2020; Joseph et al., 2021; Ruiz-Reig et al., 2023)
​	*KIF3B*	Retinal atrophy, schizophrenia, autosomal dominant ciliopathy (Adams et al., 2024; Alsabban et al., 2020; Cogné et al., 2020)	​	​	​	Plays a role in inhibiting dendritic growth and dendritic spine formation (Alsabban et al., 2020; Cogné et al., 2020; Adams et al., 2024)
	*KIF5*	Spastic paraplegia type 10 (SPG10) and axonal Charcot-Marie-Tooth disease (CMT), amyotrophic lateral sclerosis (ALS) or neonatal intractable myoclonus (NEIMY) (Cozzi et al., 2025)	​	​	Involved in nuclear translocation (Zhang et al., 2009; Olenick and Holzbaur, 2019)	Initiates the formation of outgrowths in the early stages of neurite growth, participates in the growth and patterning of dendrites, and controls the polarization of microtubules in dendrites and axons (Winding et al., 2016; Lu and Gelfand, 2017)
	*KIF7*	Brain malformations, intellectual disability, ventricular enlargement, hydrocephalus, hydrolethal syndrome, macrocephaly, CC agenesis and molar tooth sign (MTS), poor frontal lobe development, atrophy, pachygyria, heterotopia, seizures, acrocallosal syndrome, Joubert syndrome, and Al-Ghazali-Bakalinova syndrome (Kalantari and Filges, 2020; Pedraza et al., 2025)	Proliferation of apical and basal RGPs, neuronal differentiation (Dafinger et al., 2011; Ali et al., 2012; Barakeh et al., 2015; Pedraza et al., 2025)	​	​	Affects the formation of the corpus callosum (Guo et al., 2015)
	*KIF11*	Primary microcephaly (Kalantari and Filges, 2020)	​	​	Regulates the speed and direction of neuronal migration, terminating migration when the neuron reaches the CP (Falnikar et al., 2011)	Controls process retraction, neurite outgrowth branching, and dendritic spine density (Kahn et al., 2015; Wingfield et al., 2026)
	*Kif13B*	​	Promotes symmetrical divisions of RGP (Helmer and Vallee, 2023)	​	​	​
​	*KIF14*	Intrauterine growth retardation, severe microcephaly, cerebral and cerebellar hypoplasia, arginencephaly, occipital lobe/corpus callosum agenesis (Kalantari and Filges, 2020)	Participates in cytokinesis (Carleton et al., 2006; Gruneberg et al., 2006; Fujikura et al., 2013; Kalantari and Filges, 2020)	​	​	​
	*KIF15*	Braddock-Carey syndrome (BCS) with microcephaly and agenesis of the corpus callosum (Sleiman et al., 2017)	Participates in mitosis (Ostergaard et al., 2012; Sleiman et al., 2017)	​	​	Regulates the rate of axon growth and branching, prevents the penetration of plus-end MTs into dendrites (Lin et al., 2012; Xu et al., 2014; Joseph et al., 2021)
	*KIF20A*	​	Regulates the proliferation of progenitors (promotes the transition from proliferative to differentiating divisions) (Geng et al., 2018)	​	​	Inhibits microtubule sliding during neurite maturation ((Lu et al., 2013; Del Castillo et al., 2015)
	*KIF20B*	-	Participates in the formation of the midbody during cytokinesis (Dwyer et al., 2011; Janisch et al., 2013; Johnson et al., 2017; McNeely and Dwyer, 2020)	Involved in polarization and specification of processes (Johnson et al., 2017; McNeely et al., 2017)	​	Morphogenesis of pyramidal neurons, specification, growth and branching of axons and dendrites, correct orientation of apical dendrites relative to the cortical plane (Joseph et al., 2021; McNeely et al., 2017)
	*KIF21B*	Brain malformations including agenesis of the corpus callosum (ACC), microcephaly, developmental delay, and intellectual disability (Asselin et al., 2020; Narayanan et al., 2022; Tempes et al., 2020)	​	​	Affects neuronal migration (Asselin et al., 2020)	Regulates microtubule dynamics in growth cones, regulates neurite growth and branching, and affects dendritic spine density (Asselin et al., 2020; Tempes et al., 2020; Narayanan et al., 2022)
	*KIF23*	Microcephaly (Naher et al., 2023 ; 2024)	A key regulator of cytokinesis (Zhao et al., 2006; Naher et al., 2023; Naher et al., 2024)	​	​	Promotes dendritic growth (Nislow et al., 1992; Sharp et al., 1997; Joseph et al., 2021)
Myosins	Myosin II	Autism, schizophrenia, intellectual disability and neurodegenerative diseases, microcephaly, cerebral and cerebellar atrophy, deafness (Javier-Torrent and Saura, 2020; Kasza et al., 2019)	MST in primate and human corticogenesis (Wimmer et al., 2025)	​	Nuclear (but not centrosome) advancement (Bellion et al., 2005; Solecki et al., 2009; Martini and Valdeolmillos, 2010; Nakazawa and Kengaku, 2020)	​

Molecular mechanisms have been characterized only for a limited number of disorders, and the pathogenic mechanisms, as well as the specific functions of a significant number of genes associated with motor protein activity, remain incompletely characterized. They are promising candidates for study.

For example, the mechanism by which nesprin-1/2 proteins influence radial migration, as well as their role in interacting with dynein and kinesin, remains to be elucidated. Confirming one of the hypotheses–whether they function as a molecular switch or an integrating factor linking dynein and kinesin–is fundamentally important for understanding the coordination of opposing motor forces in the developing neuron. Similar gaps exist in understanding the functions of other motor proteins. In particular, the precise mechanism by which myosin II orchestrates directed nuclear movement remains to be elucidated. Another example is the dynein cofactor Ndel1, mutations in which are embryonically lethal, complicating gene inhibition experiments (Sasaki et al., 2005). For some proteins, such as NudE and Sun1/2, the clinical relevance remains to be established, as no cases of mutations in these genes in patients with neurodevelopmental diseases have been published to date.

This highlights the need for further investigation of motor protein roles in corticogenesis, including their non-canonical, non-transport functions. An integrated approach that considers both transport and non-transport functions, as well as yet-unidentified roles of these molecules, will not only deepen fundamental understanding of neurodevelopment but also establish a theoretical basis for comprehending the etiology of many nervous system diseases associated with dysfunction of these proteins (Bone and Starr, 2016; Kattuoa and Das, 2025).
